# Subtotal resection and omentoplasty of the epidermoid splenic cyst: a case report

**DOI:** 10.4076/1757-1626-2-6382

**Published:** 2009-07-21

**Authors:** Avdyl S Krasniqi, Gazmend S Spahija, Shemsedin I Hashani, Eshref A Osmani, Sejdullah A Hoxha, Astrit H Hamza, Lumturije H Gashi-Luci

**Affiliations:** 1 University Clinical Centre of KosovaRrethi Spitalit str. p.n. Prishtina, 10000Republic of Kosovo; 2 Faculty of Medicine, University of PrishtinaMother Theresa str. p.n., Prishtina, 10000Republic of Kosovo

## Abstract

**Introduction:**

Nonparasitic splenic cysts are uncommon clinical entity and because of it, there is no information regarding their optimal surgical treatment.

**Case presentation:**

A 41-years-old female with incidentally diagnosed nonparasitic splenic cyst which initially was asymptomatic. After two years of follow up, the patient underwent surgery; subtotal cystectomy and omentoplasty as an additional procedure. Postoperative course was uneventful.

**Conclusion:**

Short and mid term results showed that near total cystectomy with omentoplasty was a safe successful procedure for treatment of epidermoid splenic cyst.

## Introduction

There is confusion about the etiology, pathogenesis and classification of nonparasitic splenic cysts [1-7]. The description of these lesions still can be found as epidermoid, epithelial, “true” and “false” cysts [2,4,8]. Because these cysts are uncommon, there is no ‘evidence-based’ information regarding optimal surgical treatment [4,9].

The aim of this presentation is to report the case of epidermoid splenic cyst treated with near total cyst resection with cyst wall edges plication and omentoplasty as an additional procedure, very rarely described in treatment of nonparasitic splenic cysts.

## Case presentation

**Figure 1.**
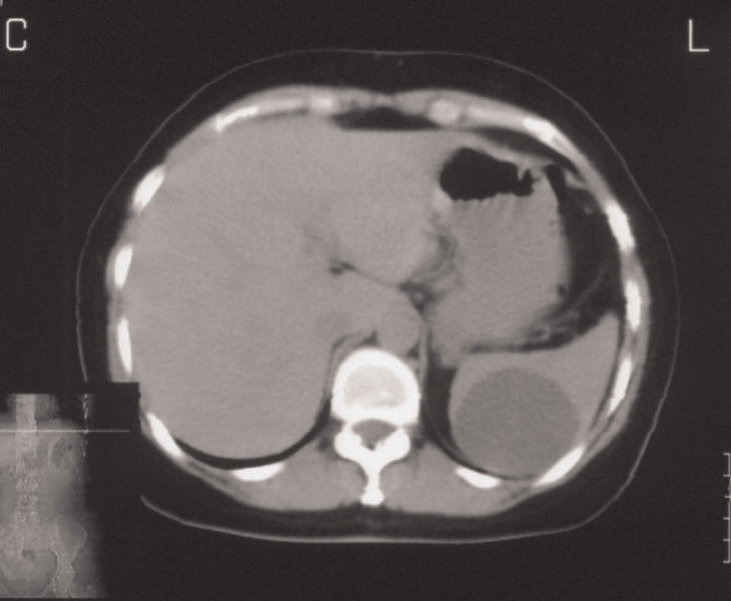
CT scan image showing a well-defined cystic lesion on the upper part of the spleen.

**Figure 2.**
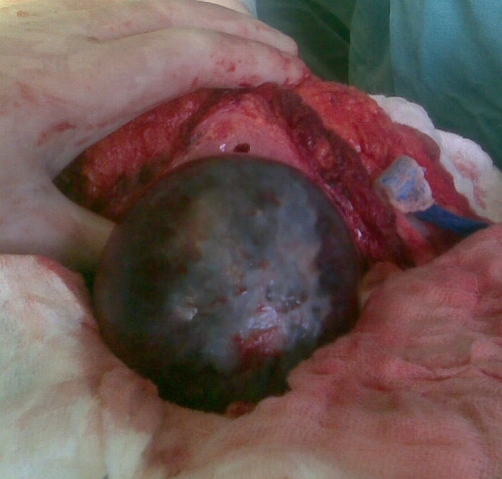
Intraoperative view showing a blue and white capsule of the nonparasitic splenic cyst.

**Figure 3.**
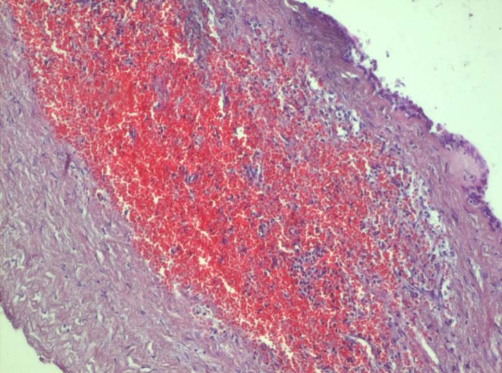
Histopathology showing epidermoid epithelium of the cystic wall (HEx 100).

**Figure 4.**
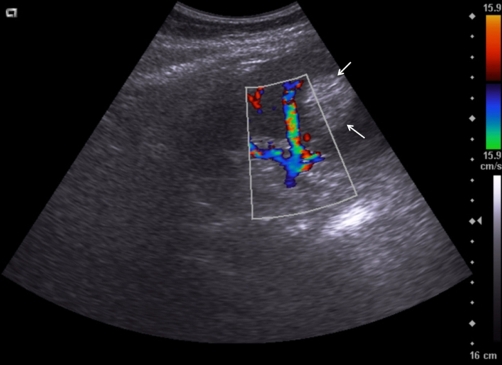
Abdominal ultrasound a month after surgery showing good vascularisation and incorporation of the omentum (between arrows) to the splenic tissue.

A 41-years-old Kosovan Albanian female with a splenic cyst diagnosed incidentally two years earlier, during the preoperative process priory to laparoscopic cholecystectomy. Initially, on abdominal sonography the splenic cyst size 4.6 × 5.0 cm was diagnosed. Since the patient comes from the endemic region for *echinococcosis*, a laparoscopic cholecystectomy was performed, whereas the splenic cyst was followed-up. But, the mild pain on the left upper abdominal quadrant continuous to persist. Repeated abdominal US and CT after one respectively two years, have showed that the splenic cyst reached the size 6.0 × 5.0 cm and 7.4 × 7.3 respectively (Figure 1). *Echinoccoccus* - antigen tests, two times were negative. The patient underwent left subcostal laparotomy. Intraoperatively, a nonparasitic cyst of upper part of the spleen was found (Figure 2). The cytology and microbiology examination of the cyst content resulted without pathologic findings. Near total excision of the cyst along with a part of pericystic splenic tissue was done; only e small part of the posterior capsule of the cyst was left. The cyst wall edges were sewn with continual PDS 3.0 sutures and the defect was covered with the omental flap - omentoplasty fixed with few resorbable single sutures. Macroscopically, typical coarse fibrous trabeculation for nonparasitic splenic cysts were present. Histologically, the diagnosis of splenic cyst containing epidermoid epithelium (Figure 3) was confirmed. Postoperative course was uneventful and the patient was discharged in good condition on the sixth postoperative day. At 5-months follow up, patient has had no symptoms. Abdominal sonography a month and four months later showed normal appearance of the remaining spleen and good incorporation of the omentum (Figure 4).

## Discussion

Symptomatic nonparasitic splenic cysts are rare entity in clinical practice. There is confusion about their etiology, pathogenesis and classification as well [1-7]. Morgenstern (2002) proposed a new classification based on characteristic gross findings [3]. A unique terminology is not yet accepted, therefore, these lesions still are described as epidermoid, epithelial, “true” and “false” cysts [2,4,8]. Epidermoid cysts are true cysts that constitute about 10-25% of nonparasitic splenic cysts [2,4]. There is no ‘evidence-based’ information regarding optimal surgical treatment, therefore, treatment modalities of these cysts are not yet clearly defined [1-14]. Cysts larger than 5 cm, as well as symptomatic and complicated cysts, should be treated. Recognition of the risk of postsplenectomy complications, especially in children, has led to spleen conserving surgery [4,8,10]. Traditionally used splenectomy, currently is attempted to be replaced with the procedures of splenic preservation. Last years, the most often performed laparoscopic and open spleen preserving procedures in treatment of nonparasitic cysts were partial splenectomy and near total cystectomy - cyst “decapsulation” [3-9]. Laparoscopic approach has gained increasing acceptance in splenic surgery. But, although there are reports that presents good results with laparoscopic excision of nonparasitic splenic cysts, the largest retrospective studies [7,9,15] showed that laparoscopic treatment is associated with a high recurrence rate. Fisher *et al* [2008] have reported a recurrence rate of 71% [9]. In a recent report, Palanivelu *et al.* (2008) concluded that plication of the cyst wall edges prevents the cyst walls from adhering and causing recurrence, as well as helping to control hemorrhage [7]. Although there are no long-term follow-up results, based on short and mid-term follow-up, both main spleen preserving procedures such as partial splenectomy and near total cystectomy are acceptable. Nowadays, spleen-preserving techniques should be attempted in every case of splenic nonparasitic cysts, therefore, surgeons must master both techniques [3,5,7,10].

In our presenting case, through laparotomy was performed near total excision of the cyst along with a part of pericystic splenic tissue. Based on our long-term experience in treatment of hydatid cysts [16], the remained cavity and cyst wall edges were cared with continual absorbable sutures and omentoplasty. Omentoplasty as an additional procedure to splenic subtotal cystectomy, in English literature, was described only in few reports [17,18]. Short and mid term follow up showed that this was a safe procedure, and we think that the additional wall edges sutures and omentoplasty could have a positive effect in controlling hemorrhage, prevention of recurrence and perisplenic adhesion formation as well. However, this need to be analyzed in larger groups and long-term follow up.

## Abbreviations

CT: Computerized tomography
PDS: Polydioxanone sutures
US: Ultrasound scan
